# Personal

**Published:** 1902-10

**Authors:** 


### PERSONAL.

Dr. V. M. Griswold, who some years ago removed from Fredonia, N. Y., to Westerly, Rhode Island, has returned to his former home and resumed his professional work at Fredonia.

---

Dr. Frederick Peterson, of New York, formerly of Buffalo, recently spent a few days in this city in company with his mother, Mme Peterson-Berg.

---

Brigadier General William H. Forwood, surgeon-general United States Army, having reached the statutory age, was retired September 7, 1902. Brigadier-General Robert M. O'Reilly assumed the duties of Surgeon-General, September 8, under an appointment made by The President several weeks ago.

---

Dr. Sydney A. Dunham, of Buffalo, announces the removal of his offices to 239 Delaware Avenue, second door above Chippewa Street. Hours: 1 to 4 and 7 to 8 p.m. Telephone, Tupper 262. *X*-Ray examinations.

Dr. J. M. O'Neill, of Buffalo, has removed from 536 Swan Street to 381 Swan Street. Hours: 8.30 to 9.30 a.m., 1.30 to 3 p.m., and 6.30 to 9 p.m. Telephone, Howard 212.

Dr. Maurice J. Lewi, of New York, announces that hereafter he will limit his practice to diseases of the foot. Office and residence, 149 W. 85th Street. Hours: 9 a.m. to 4 p.m. Telephone, Riverside 2059.

Dr. A. W. Bayliss, of Buffalo, who with Mrs. Bayliss, has been spending the summer in Europe, returned September 18, 1902, and resumed his professional work at 592 Spring Street.

Dr. Alice E. Rowe, of Springfield, Mass, has been appointed assistant physician at the Gowanda State Hospital for the Insane. She was graduated at the Boston University Medical School in 1893 and afterward spent a year in the Massachusetts Homeopathic Hospital. Dr. Rowe has made a special study of nervous diseases and spent four months of last year studying in the hospitals in Berlin and other places in Europe. She holds the state license to practise, and has passed the civil service examination.

Johannes Orth, professor of pathological anatomy in the University of Göttingen, succeeds the late Professor Virchow to the chair of pathological anatomy in the University of Berlin. Professor Orth was for many years Professor Virchow's assistant.

Dr. William H. Mansperger of Buffalo, has been appointed chief surgeon for the Erie Railway vice Dr. C. M. Daniels, deceased.

The following-named physicians who have been touring in Europe during the summer have returned and resumed their professional work: Drs. Max Breuer, Roswell Park, Allen A. Jones, Alexander McNeil Blair, Julius Pohlman.

